# Fingerprint of Droplet Shape and Vortex in the Line Shape at the Electronic Band Origin of Phthalocyanine in Superfluid Helium Droplets

**DOI:** 10.1021/acsphyschemau.5c00018

**Published:** 2025-08-07

**Authors:** Rupert P. M. Jagode, Alexander Scrimgeour, Florian Schlaghaufer, Johannes Fischer, Alkwin Slenczka

**Affiliations:** Institute for Physical and Theoretical Chemistry, 9147 University of Regensburg, 93053 Regensburg, Germany

**Keywords:** microsolvation, superfluid helium nanodroplets, vortex, phthalocyanine, electronic spectroscopy

## Abstract

X-ray and XUV diffraction experiments have visualized both the outer shape and quantum vortices inside individual superfluid helium droplets. Both features are effective on the helium induced signature observed as the spectral shape and position of the electronic transitions of molecules doped into helium droplets. In this article the helium induced signature at the electronic band origin of phthalocyanine is re-examined systematically comprising previous analytical results as well as newly reported experimental investigations. Helium-induced effects such as a nonmonotonous evolution of the solvent shift and the emergence of an optical anisotropy, both observed for rather large helium droplets, are the spectroscopic response on the analytical results reported from diffraction experiments. All helium induced spectroscopic features can be explained as an expression of London dispersion interaction under the varying structural conditions of helium droplets.

## Graphical abstract

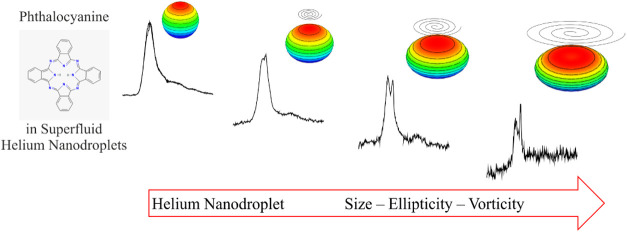

## Introduction

Investigation of structure and dynamics of molecules, molecular aggregates and clusters is greatly facilitated by careful preparation of such species. In this regard, superfluid helium nanodroplets comprise a uniquely gentle host.^1^ Nevertheless, it releases its fingerprint that needs to be separated from the dopant signals.^2^ Whether designing of clusters^3–9^ or molecular aggregates^10^ or investigating dynamic processes^10–13^ including fundamental chemical reactions^14^ or dealing with large biomolecules^15^ the impact of the helium environment has to be considered. Of particular sensitivity to the helium environment is electronic spectroscopy of the dopant species.^2,16,17^ The investigation of electronic spectra of molecules in superfluid helium nanodroplets can have different purposes. Either the superfluid helium droplet is simply a vehicle for cooling molecules with the aim of facilitating the analysis of molecular spectra.^4,18–20^ Alternatively, the dopant molecule is used as a kind of sensor that provides information about the superfluid helium droplet.^17,21–24^ However, the two purposes are intertwined, so that spectra of molecules in superfluid helium droplets always carry signatures of both the molecule and the helium droplet at the same time. In order to exploit the potential arising from the spectroscopy of molecules in superfluid helium droplets, these two contributions are to be disentangled. Despite numerous corresponding investigations this is still an unsolved problem that will further be discussed on the example of phthalocyanine in superfluid helium nanodroplets.

New spectroscopic data confirm previous line shape analyses at the electronic band origin of phthalocyanine (H_2_Pc) doped in superfluid helium droplets^25,26^ and in addition clarify features that could not be explained so far.^27^ The clarifications are motivated by recent reports on the shape and internal structure of superfluid helium droplets as obtained from X-ray and XUV-diffraction experiments.^28–34^ Accordingly, spectral signatures in electronic transitions of phthalocyanine that are clearly identified as helium-induced can now be attributed to properties of helium droplets concerning their shape and internal structure. In general, the variation of the spectral shape at the electronic band origin of H_2_Pc with the size distribution of helium droplets is an expression of London dispersion interaction. As explained in refs 25,26 a key to quantitative simulation of the line shape is considering the correct droplet size distribution which is not that, generated by the droplet source but instead only those which were doped with a single H_2_Pc molecule. This paper reports on features such as line splitting and a reversal of the dependence of solvent shift on droplet size^27^ which are fully in line with London dispersion forces as the droplet shape and the presence of vortices are considered.

From the very beginning of molecular spectroscopy in superfluid helium droplets, inhomogeneous line broadening due to the droplet size distribution was obvious.^1,35^ Among the various spectroscopic techniques, electronic spectroscopy stands out in the sensitivity to the helium environment.^2,11,16,17,36^ It results from the dopant to helium interaction, that at short-range is dominated by the repulsion between helium and the dopant’s valence electrons, and in the large range by the attractive part of London dispersion interaction. Of course, valence electrons are those involved in the electronic transition. H_2_Pc (Tetrabenzotetraazaporphyrin) is a closed shell planar organic molecule and, due to only two instead of four inner hydrogen atoms, a slightly asymmetric rotor.^37–39^ Its interaction with helium is adequately described by the London dispersion forces, which result in a rather rigid helium double layer (cf. Figure 1) as revealed by path integral Monte Carlo simulations.^40^ Further into the droplet volume the attractive component dominates whose potential energy is proportional to r^–6^. Thus, the impact of helium embedding on a dopant varies with the amount of helium tantamount to the droplet size. And due to the r^–6^ dependence, London dispersion is effectively limited to a finite range. The perturbation on the dopant by the surrounding helium therefore accumulates with increasing droplet size and finally converges to a maximum value as the droplet radius reaches or exceeds the effective range of London dispersion forces. The effective range is not only dopant specific but in addition specific to a molecule’s quantum state which is the reason for the solvent induced spectral shift of resonance frequencies. Beyond this range the variation of the droplet size does not effect the dopant anymore. Thus, the resulting solvent shift of electronic resonance frequencies increases monotonously with the droplet size and finally approaches a limiting value. Since the helium droplet source generates droplets at a certain size distribution, possibly including sizes below the limiting range of dispersion interaction, electronic transitions suffer inhomogeneous line broadening. As the droplet size distribution is shifted to larger droplets this kind of inhomogeneous line broadening decreases and finally vanishes gradually and will be absent for any size distribution beyond the effective reach of London dispersion forces. Accordingly, a model was developed to calculate the solvent shift of the electronic resonance frequency of the dopant, which results from the London dispersion interaction integrated over the droplet volume. Considering the appropriate droplet size distribution, i.e., the part of the pristine size distribution that is singly doped with H_2_Pc, the helium-induced inhomogeneous line broadening of the experiment could be simulated quantitatively.^25,26^

**Figure 1:**
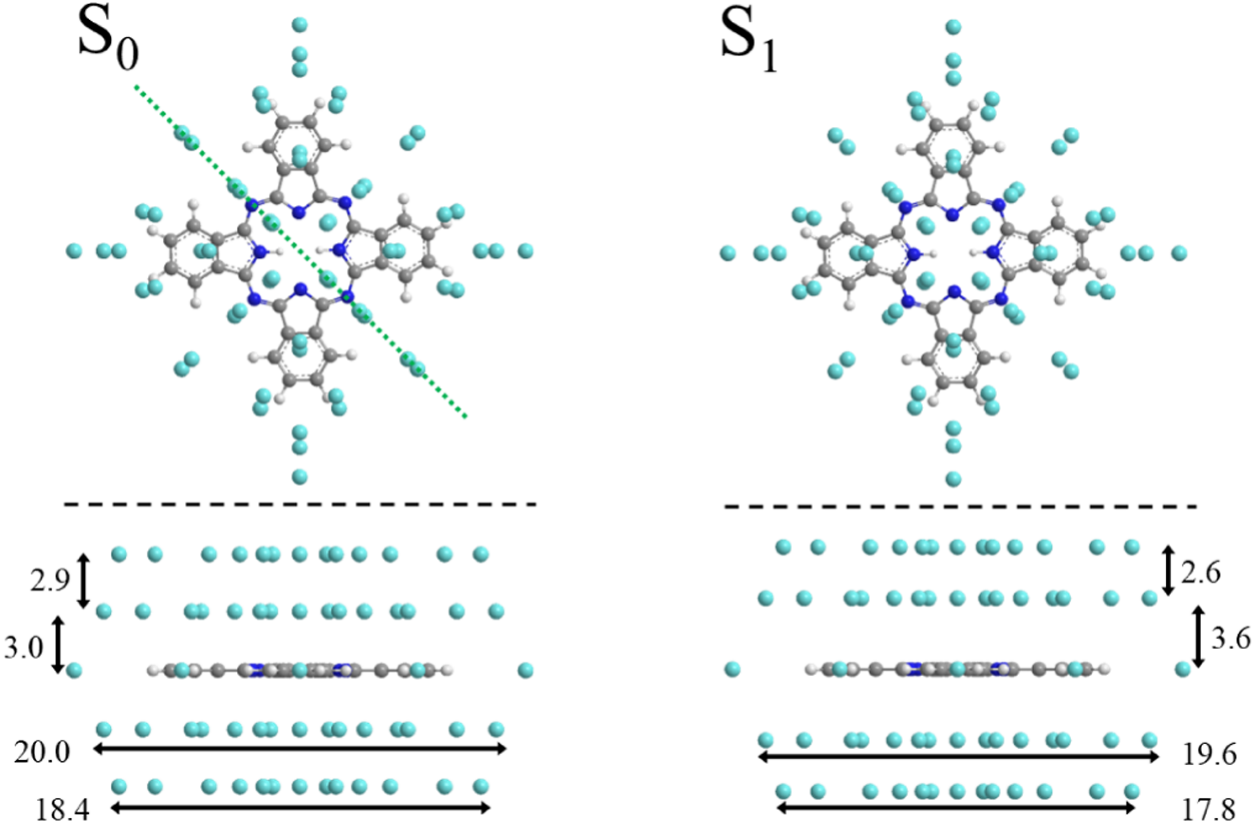
Structure of the phthalocyanine helium solvation complex with two layers of helium on each face. The geometric parameters of the helium layer are added in units of Å for the electronic ground state S_0_ (left) and the first electronically excited state S_1_ (right) in top view (top) and side view (bottom), the latter along the green line.

For average droplet sizes in the order of 10^7^ helium atoms assumed to be beyond the effective reach of the London dispersion interaction, the spectral shape at the electronic band origin was found split to a double peak with an overall half-width smaller than the singly peaked inhomogeneously broadened line shape.^23^ In line with the expectation of vanishing inhomogeneous line broadening, the double peak was interpreted as rotational band contour within the electronic transition of H_2_Pc in helium droplets (cf. Figure 8 in ref 23). According to an empirical rule of thumb—moments of inertia of molecules doped in helium droplets increase by a factor of 3 ^41,42^ —the experimental spectrum of H_2_Pc in such large helium droplets could be quantitatively simulated by the correspondingly adjusted rotor. For simplification H_2_Pc in helium droplets was approximated by an oblate symmetric top rotor. In summary of refs 23,25,26 , London dispersion interaction appears to be the key quantity responsible for solvent shift and, as a consequence of a droplet size distribution, for inhomogeneous line broadening that vanishes for droplet sizes larger than the effective reach of the dispersion interaction. Henceforth, his model will be called dispersion model.

The validation of the dispersion model for inhomogeneous line broadening, as justified by the work cited above, ignores two crucial aspects. First, the data considered leave a gap in the range of droplet sizes from 10^5^ to 10^7^ helium atoms. Second, the double peak signal undergoes a slight reversal of the solvent shift on the order of 10^–2^ cm^–1^ after it reached a maximum for smaller average droplet sizes. Such a reversal is not comprehensible according to the dispersion model.^25,26^

Obviously, within the size gap the solvent shift undergoes a reversal despite increasing average droplet sizes. Recent investigations^27^ within this size gap revealed a rather complex development of the line shape, however, confirmed the formerly reported double peak signal.^23^ A purely empirical analysis of the line shape using Gaussian peaks required three contributions, one of which appeared to be almost transient within the range of droplet sizes considered. Besides the reversal in the solvent shift, a multiply peaked intensity profile that varies significantly with the effective droplet size distribution speaks against a rotational band contour of a freely rotating H_2_Pc-helium solvation complex. Such a complex shown in Figure 1 results from the short-range part of the dopant to helium interaction,^40^ which should not be influenced by changes in the size of rather large droplets. Apart from this, the simulation presented in ref 23 was calculated for a planar symmetric top rotor. As shown in Figure 1 corroborated from ref 40, the solvation complex is not planar.

Herein, we report a revised interpretation for the development of the line shape at the electronic band origin of H_2_Pc in superfluid helium droplets which includes the full range of droplet sizes accessible with the Göttingen helium droplet source.^1,36,43–45^ Revision concerns in particular the features observed in combination with line splitting in larger helium droplets. The new interpretation is fully in line with the knowledge on shape and structure of superfluid helium droplets gained from recent X-ray and XUV-diffraction experiments.^28–31^ Peak splittings as well as solvent shift reversal are recognized as expression of dispersion interaction, however, under consideration of particular droplet features so far not considered in the dispersion model.

## Experimental Section

The experimental equipment is almost identical to that described in previous work from our laboratory.^27^ It consists of a vacuum machine with two differentially pumped vacuum chambers. The first chamber contains the helium droplet source which is a copy of the continuous flow nozzle developed in Göttingen.^35^ It is equipped with a platinum orifice of 5 μm in diameter. The nozzle is attached to a Sumitomo cold head RDK-408D2 and compressor unit CSW-71 which provides cooling of the nozzle under gas flow down to about 6.0 K. Vacuum of 2·10^–8^ mbar is accomplished by a 2200 L/s turbo molecular pump (Pfeiffer TPH 2200) backed by a mechanical booster pump (ULVAC PMB-006CM) and a rotary vane pump (Pfeiffer DUO 060 A).

The second vacuum chamber is evacuated to about 2·10^–8^ mbar by a turbo molecular pump (Pfeiffer Hipace 300) backed by a rotary vane pump (Pfeiffer DUO 030 A). It contains the pick-up unit for doping of the helium droplets and the fluorescence detection unit and is accessed by the helium droplet beam via a conically shaped skimmer with an opening of 1.4 mm in diameter. Measured from the nozzle, the distance to the skimmer is about 20 mm and to the pick-up unit about 120 mm. The pick-up unit consists of a stainless steel cylinder surrounded by a heating wire. It is about 30 mm in diameter and 20 mm high. The heating wire is shielded by a tight-fitting stainless steel cover and additionally by a copper cylinder which is contacted to a Dewar flask filled with liquid nitrogen. In Figure 2 a heating power to temperature curve is shown. However, for reasons of the contact spot of the thermocouple the real sublimation temperature may have been about 20 up to 30 K higher. Additional 80 mm behind the pick-up unit a laser beam intersects the helium droplet beam at right angles. Orthogonal to both beam axes a condenser lens (*f*# = 2) collects the laser-induced fluorescence which is imaged onto the photocathode of a photo multiplier tube (PMT) (Hamamatsu R943–02), which can be shielded by an appropriate edge filter in order to eliminate laser stray light. For the present study, the detector was operated without an edge filter. The PMT signal is amplified (two stages of Stanford Research Systems SR445) and fed into a photon counter (Stanford Research Systems SR400). A quadrupole mass spectrometer (Leybold TSP TH 300) is mounted on axis to the helium droplet beam at the back flange of the second vacuum chamber. It allows to check the operation of the helium droplet beam as well as the background gas in the detection chamber. In contrast to our second helium droplet machine operated with oil diffusion pumps the current machine warrants for oil free vacuum which is critical upon working with large helium droplets. The background gas consists almost exclusively of water accompanied by minor contributions of nitrogen and oxygen. Under operation, ionized fragments of H_2_Pc and of helium clusters add in.

**Figure 2:**
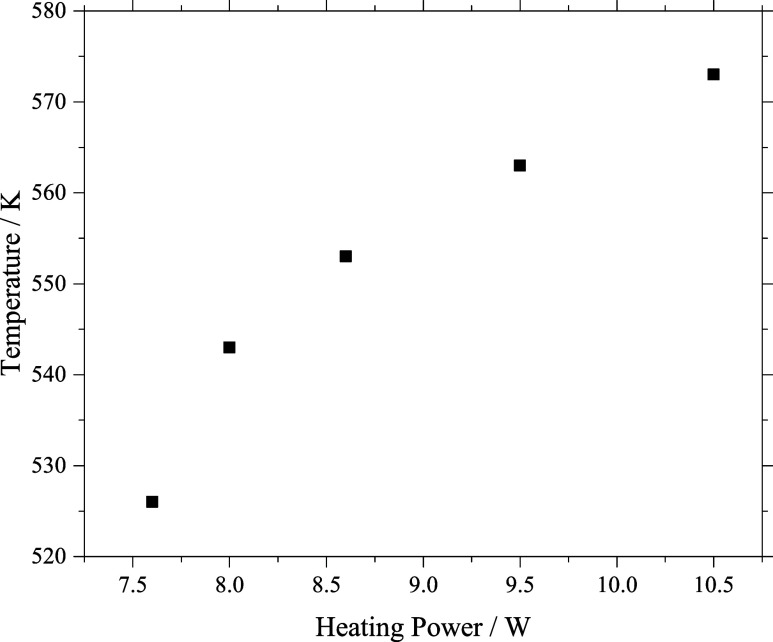
Temperature of the pickup oven as a function of the heating power. Note that the temperature values are a lower limit and the real sublimation temperature may be 20 or 30 K higher.

The laser system is an actively stabilized single mode ring dye laser (Coherent 899–29 Autoscan) with a bandwidth of less than 1 MHz. It is pumped by 10 W @ 532 nm from an optically pumped semiconductor laser (Coherent Verdi G10) and operated with DCM dye. The output power of the dye laser operated in single mode peaks at around 0.5 W. In most of the experiments the laser is attenuated to only 1% of the full power in order to avoid saturation broadening. The laser beam was guided to the vacuum machine by a single mode quartz fiber. The transmission through the fiber imprinted slight ellipticity to the otherwise linearly polarized laser beam and a rotation of the main polarization axis. Before entering the vacuum machine, the linear polarization was restored by a polarizer (Glan-Thompson prism), and a double Fresnel rhombus was used for continuous rotation of the polarization plane. In order to ensure coupling into the vacuum machine independently of the polarization plane, the input Brewster window was replaced by a flat quartz window.

Frequency stepping of the laser and photon counting is synchronized by hardware hand-shaking between the laser and the photon counter. The management of the data reading and storing is accomplished by computer control via homemade software.

## Results

Since our first line shape investigation at the electronic band origin of H_2_Pc in superfluid helium droplets^25,26^ we have frequently remeasured this line shape. Recently, the gap in the size range of helium droplets from 10^5^ up to 10^7^ that was not explored in refs 23,25,26 was filled.^27^ It needs to be mentioned that helium droplets in the range of sizes below 10^5^ atoms are generated from gas phase expansion while larger droplets stem from expansion of liquid helium.^36,43–45^ While line shapes recorded from gas phase expansion were perfectly reproducible by means of the dispersion model^25–27^ this could not be established for those recorded from the expansion of liquefied helium. In particular, the line splitting and the solvent shift reversal as described for the first time in ref 27 was not expected from the dispersion model. Therefore, a systematic handling of the experimental parameters tuning the effective droplet size distribution was of interest in order to clarify the problem of reproducibility and, of course, all the accompanying line shape features. Basically, there are two experimental processes that determine the effective droplet size distribution which is the fraction that enters the detection volume as singly doped droplets. The first process is the generation of droplets from the droplet source that comes with a characteristic size distribution that was accurately explored as summarized in the following three seminal refs 36,43,44 . Whichever size distribution, it shifts to larger sizes as the stagnation pressure increases and/or the nozzle temperature decreases. The second process is the doping of the droplets on the flight through a pick-up unit. With increasing particle density in the pick-up unit, the fraction of singly doped helium droplets shifts to smaller droplets and vice versa. The particle density varies with the sublimating temperature which follows the heating power applied to the pick-up oven (cf. Experimental chapter Figure 2).

In Figure 3 a map of 50 panels shows spectra of the electronic band origin of H_2_Pc in helium droplets, measured with decreasing nozzle temperature (from top to bottom), as indicated on the left, and with decreasing heating power at the pick-up oven (from left to right), as indicated at the bottom of the figure. Within the spectral window of only 0.7 cm^–1^ no other signals are expected neither from clusters of H_2_Pc with impurities picked up from the background gas nor from (H_2_Pc)_*n*_ aggregates. Each panel shows two spectra measured consecutively to confirm stationary experimental conditions. Each column was recorded for constant heating power quantified at the bottom while each row was recorded for constant nozzle temperature marked on the left. The stagnation pressure of helium in the droplet source was kept constant at 20 bar. Thus, the upper left spectrum was recorded for smallest effective droplet sizes while the bottom right spectrum was recorded for the largest. According to droplet size investigations^36,43–45^ the experimental conditions for the droplet source collected in this map consider average droplet sizes from 10^4^ to close to roughly 10^8^ helium atoms. The spectrum recorded for 7.0 K nozzle temperature and a heating power of 8.6 W is almost identical to that reported in ref 23. For reduced heating power (same row to the right) single molecule doping is shifted to larger droplets within the size distribution delivered for 7.0 K and 20 bar as expressed by the changing spectral shape of the double peak. Even though the effective droplet size distribution may not cover the full range of the nascent size distribution from the droplet nozzle, a reduction of the heating power at the pickup oven and/or a reduction of the nozzle temperature cause a shift of the effective size distribution to larger droplets which causes gradual changes in the spectral line shape.

**Figure 3:**
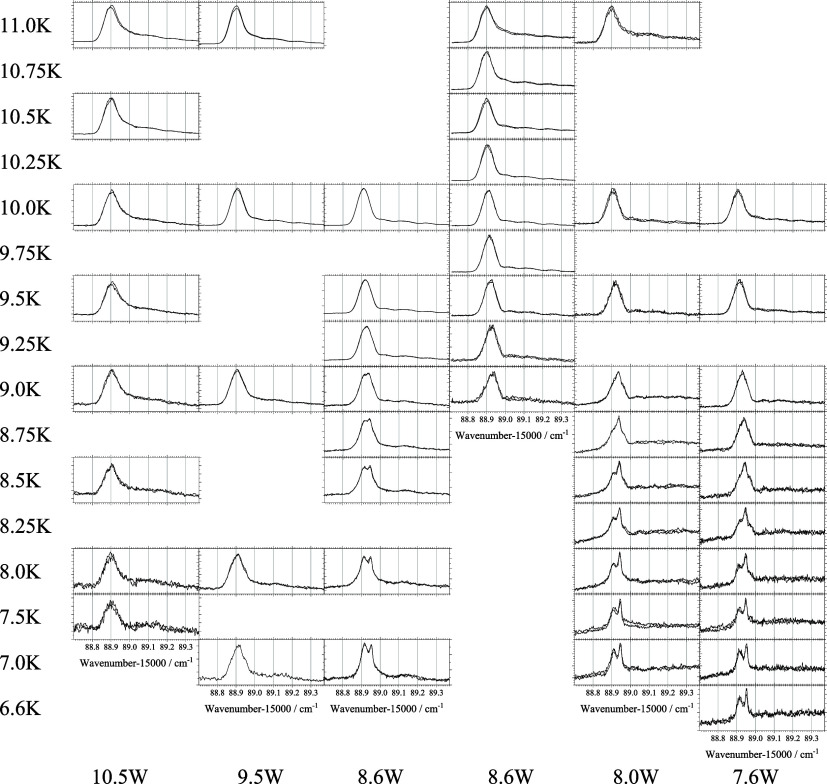
Electronic band origin of a single H_2_Pc molecule doped into helium droplets recorded for a stagnation pressure of 20 bar and decreasing nozzle temperatures as indicated on the left side from top to bottom and upon decreasing heating power at the pick-up oven as indicated on the bottom line from left to right. When going from top to bottom and from left to right the average size of singly doped helium droplets increases. Abscissa are scaled identical for all 50 spectra. Ordinate was scaled individually to the peak maximum. Each panel shows two successively recorded spectra to check for stationary conditions.

The major message from the map of spectra shown in Figure 3 is the presence of a singly peaked line shape for the small droplet sizes (heating power 10.5W) that transforms into a split line shape for large droplet sizes (the lower right quadrant of the map). For doping optimized for the smallest droplet sizes under consideration (10.5 W and 11.0 K) a tuning of the nascent droplet size distribution to larger droplets by decreasing of the nozzle temperature (left column 10.5 W and 11.0 K down to 6.6 K) remains almost ineffective on the spectral shape of the electronic band origin. Similarly, for the smallest nascent droplet sizes a tuning of single particle pick-up to larger droplets by decreasing of the heating power (*T* ≥ 9.5 K and heating from 10.5 W down to 7.6 W) a singly peaked and asymmetric line shape is maintained, throughout. Consequently, in both cases the effective droplet size distribution, namely those which are singly doped with H_2_Pc, remains safely below 10^5^ helium atoms. The larger droplets generated at lower nozzle temperatures within the left column do not contribute to the recorded signal due to mainly cluster formation as consequence of multiple doping with H_2_Pc. This series is in addition a proof for the absence of cluster signals whether H_2_Pc_*n*_ aggregates or H_2_Pc with impurities that would overlap with the spectral range under investigation. Within the top row, larger droplets are simply not present so that the reduction of the heating power causes mainly a reduced amount of singly doped out of the small droplets instead a shift to larger sizes. Since H_2_Pc is the only chromophore for the deep red excitation wavelength, there are only its aggregates or clusters with impurities that might contribute via fluorescence. However, spectral coincidence of electronic transitions within a tenth of cm^–1^ can safely be excluded for such complexes.

At the given stagnation pressure of 20 bar a deviation from a singly peaked asymmetric line shape is only recognized for nozzle temperatures below 9.0 K. However, already above 9.0 K and for heating powers at and below 9.5 W this single peak suffers a reversal of the solvent shift. Despite increasing effective droplet sizes, the line shape does not shift further to the red or converges to a maximum in the red shift as to be expected from the dispersion model. Instead, a reversal to smaller red shifts is observed that maintains also for multiply peaked spectra.

The so-called triple peak splitting addressed in ref 27 could be reproduced qualitatively and is most clearly seen between 9.0 and 8.0 K recorded for a heating power of 7.6 W (sixth column). This particular profile is an intermediate feature that for further increasing droplet sizes (down the column) develops to the double peak qualitatively in agreement with refs 23 and 27. For a nozzle temperature of 7.0 K and below, the double peak is present for heating powers of 8.6 W and below. The variations in the intensity profile within this doublet pretend to deal with a droplet size induced effect. However, as will be shown below, the polarization of the laser is actually a major parameter that is responsible for the fluctuations in the intensity profile. In the following, the two peaks are referred to as blue peak and red peak for the higher and lower wavenumber position, respectively.

The polarization of the laser has been ignored so far, as previous studies have shown completely identical line shapes, regardless of which polarization plane was chosen for the laser.^46^ However, these polarization studies had been performed for the singly peaked slightly asymmetric line shapes and, thus, for droplet sizes below 10^5^ helium atoms. Upon testing, a significant optical anisotropy was found for the double peak spectra. As an example, Figure 4 shows spectra recorded for a stagnation pressure of 20 bar, a nozzle temperature of 8 K (a and b) or 7 K (c and d), and a heating power of 7.6 W. For s-polarization (a and c), the red peak dominates, whereas with p-polarization (b and d), the blue peak dominates. This kind of optical anisotropy was found to be qualitatively identical for all split spectra. Thus, the differences in the double peak intensity profile as depicted in the spectra map of Figure 3 is also due to different orientation of the plane of polarization of the laser, which was not controlled for these spectra. Upon daily realignment of the single mode quartz fiber the state of polarization of the transmitted laser beam was erratically varied. In the data map of Figure 3, the polarization state of the laser was fairly constant within each column, but rather different within each row. Corresponding data from ref 27 have been recorded for laser polarization perpendicular to the plane of droplet and laser beams.

**Figure 4:**
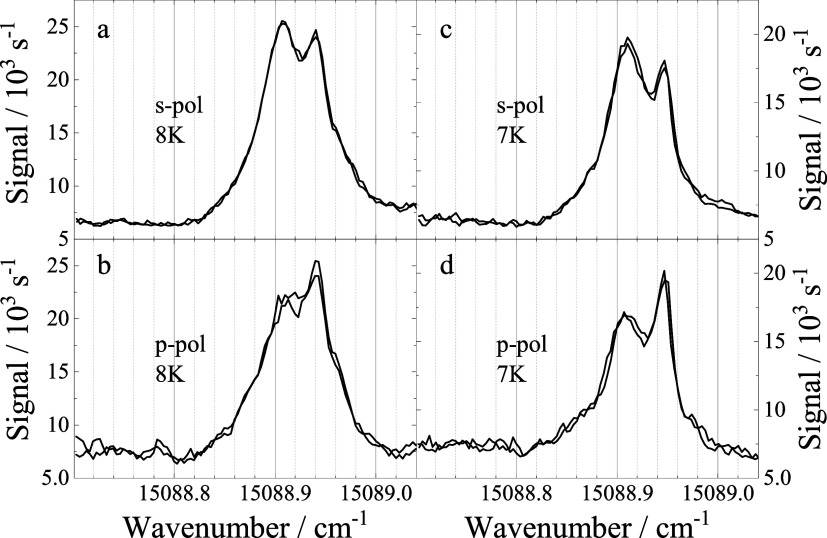
Spectra of the electronic band origin of H_2_Pc in helium droplets recorded for a stagnation pressure of 20 bar and a nozzle temperature of 8 K (a, b) and 7 K (c, d), and a heating power of 7.6 W. These spectra were recorded for s-polarization (a, c) and p-polarization (b, d) of the laser with respect to the plane of droplet beam and laser beam. Each panel shows two spectra recorded under identical conditions to check whether the experimental parameters were stationary.

Obviously, optical anisotropy is only present for droplet sizes above 10^5^ helium atoms that are generated from the expansion of liquid helium. Both the optical anisotropy and the variation of the double peak intensity profile within each column in Figure 3 suggests the presence of only two signal components instead of three as insinuated by the empirical triple Gaussian analysis in ref 27. One component is the spectrally rather sharp peak on the blue side of the doublet that is assumed to reside on the blue tail of an asymmetric and spectrally much broader signal peaking at the red side of the doublet as second component. As examples, three spectra are replotted in Figure 5 that were recorded for a heating power of 7.6 W (right column in Figure 3) at nozzle temperatures of 8.75 K (a), 7.5 K (c), and 6.16 K (e). Panels a, c, and e show the spectra (black line) amended by a smoothed version which was manually extrapolated (red line) to separate the sharp spike. The difference spectra are plotted in panels b, d, and f (black line). Each was amended by Gaussian line fit (blue line). Within each row of panels in Figure 5, the scaling of the ordinate is identical, while the abscissa is identical for all six panels. This shows that the intensities of the two signal components react differently to the change in the effective droplet size distribution. Attempts to fit a Poisson-intensity profile of H_2_Pc_*n*_ aggregates have failed.

**Figure 5:**
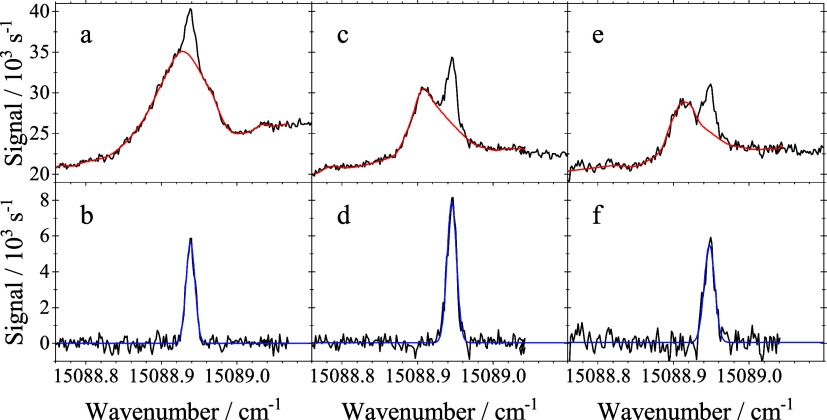
(a, c, e): Black line: Electronic band origin of H_2_Pc in helium droplets recorded for heating power of 7.6 W, a stagnation pressure of 20 bar, and a nozzle temperature of 8.75 K (a), 7.5 K (c), and 6.16 K (e). Red line: Smoothed spectrum with extrapolation to isolate the sharp peak. Scaling of the ordinate is identical for all three panels. (b, d, f): Black line: Difference spectra of the black and red lines in the top panels. blue line: Gaussian fit. Scaling of the ordinate is identical for all three panels. Scaling of the abscissa is identical for all six panels.

As result of a corresponding signal analysis for all 50 spectra shown in Figure 3, the peak positions are plotted in Figure 6 as a function of the droplet source temperature. The five symbols represent various heating powers as listed in the legend. Data for source temperatures at and above 11 K (half filled pink circles) were taken from ref 27 which show single peaked spectra that follow the dispersion model reported in refs 25,26 . Falling below 11 K, the reversal in the solvent shift is clearly visible. Falling further below 9 K the development of the peak splitting with two signal contributions, namely, the sharp blue peak (upper trace) and the red asymmetric peak of the remaining fraction (lower trace) is shown. The vertical bars added to the upper trace represent the width of a Gaussian line fitted to the signal component. Corresponding data for the second asymmetric signal component exceed the range of the ordinate in Figure 6 by far. It should be noted that these data were recorded for a helium stagnation pressure of 20 bar. The effect upon increasing the stagnation pressure would mainly cause a shift of these peak positions to higher temperatures (to the right within the diagram of Figure 6).

**Figure 6:**
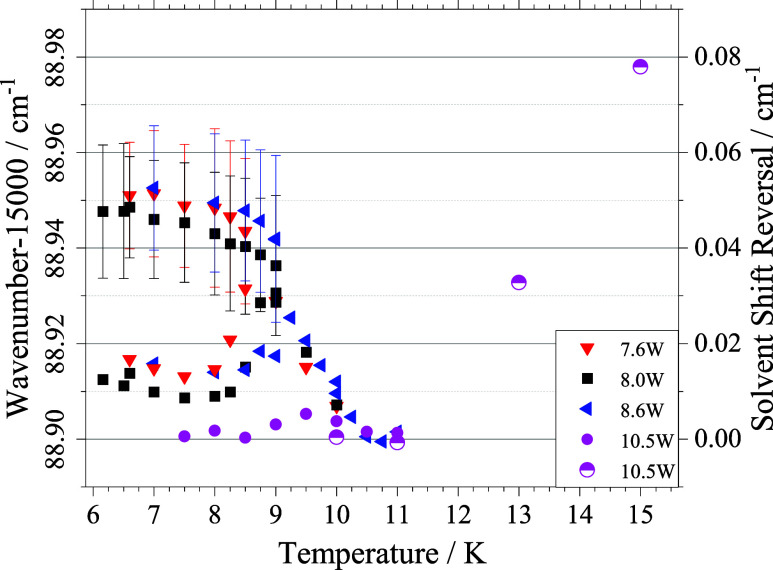
Spectral peak position (left ordinate) as a function of the droplet source temperature as obtained for different heating powers at the pick-up oven that is indicated by the symbols assigned in the legend. Right ordinate is scaled to the maximum observed red shift and represents the solvent shift reversal. Vertical bars at the upper trace below 9 K are the line width of the Gaussian fit to the sharp peak (cf. Figure 5). Corresponding values for all other peak positions exceed the range of the ordinate by far.

## Discussion

The new data presented in Figure 3 and in addition their optical anisotropy depicted in Figure 4 explains the problem with the reproducibility of such spectra. While the line splitting at the electronic band origin was unquestionable, the reproducibility of the intensity profile posed a problem. It is now obvious that the variation in the intensity profile of multiple peaked spectra as depicted in Figure 3 is not only a matter of effective droplet size distribution but in addition a matter of laser polarization (cf. Figure 4). In our previous experiments reported in ref 27 laser polarization was kept fixed perpendicular to the plane of droplet and laser beam. And for spectra shown in Figure 3 polarization of the laser in the vacuum machine had been erratically rotated and delinearized by transmission through a single mode quartz fiber (cf. Experimental Section). For investigating optical anisotropy, perfect control over the laser polarization was achieved by means of a linear polarizer and a double Fresnel rhombus. As a result of the optical anisotropy, all split spectra were recognized as consisting of two independent signal components, which also differed significantly in spectral shape and in their response to the droplet size distribution (cf. Figure 5). Consequently, an assignment of the double peak to a rotational band contour of a H_2_Pc-helium solvation complex as proposed in ref 23 can safely be excluded. The structure of the solvation complex and, thus, its rotational band spectrum is expected to stay constant under variation of the droplet size.

The sharp Gaussian peak has a constant spectral width of Δν = 0.013(1) cm^–1^, which is certainly below the spectral width of the rotational band contour presented in ref 23 (Δν = 0.1 cm^–1^). Rotational constants of the H_2_Pc-helium solvation complex shown in Figure 1 as deduced from quantum chemical calculations for ref 40 are listed in Table 1.^47^ To simulate the corresponding rotational band spectrum, the rotational constants of the excited state, which are added to Table 1, were chosen according to the following considerations. First, the distance of the first layer of helium had to be increased in order to account for the change in electron density distribution upon electronic excitation of H_2_Pc. Furthermore, the gap to the second layer was slightly reduced that shifts the resulting helium density within the two layers toward that of superfluid helium (cf. Figure 1). As depicted in Figure 7 the resulting half width of the rotational band amounts to about Δν = 0.06 cm^–1^ which clearly exceeds the spectral width of the sharp Gaussian signal component. One may argue about the changes in the rotational constants (−4% for A and B and +3% for C) upon electronic excitation. However, the chosen values reveal the spatial increase of the density distribution of the valence electrons upon electronic excitation whose effect on the moments of inertia is significantly amplified by the attached helium layers. Even for half the percentage changes in the rotational constants, the spectral width of the rotational band contour still exceeds that of the sharp Gaussian peak identified in the experimental spectra. However, the residual red peaked asymmetric signal component (signal without the Gaussian peak, cf. Figure 5a,c,e) exceeds the width of the rotational band contour (cf. Figure 6) so that this signal contribution could be fitted by a convolution of the simulated rotational band spectrum with an appropriate helium induced line broadening function.

**Figure 7:**
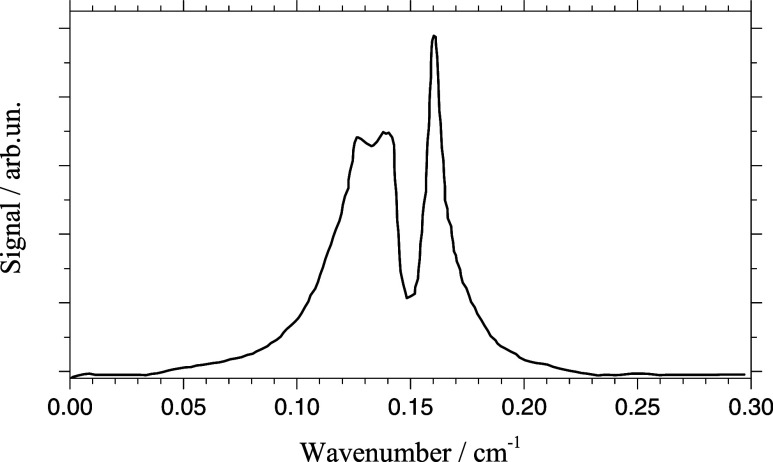
Rotational band spectrum at the electronic band origin of a H_2_Pc-helium solvation complex at a temperature of 0.37 K according to the rotational constants listed in Table 1 and as depicted in Figure 1 (cf. ref 47).

**Table 1:** Rotational Constants in Units of 10^–5^ cm^–1^ as Obtained from a Gas Phase Spectrum of Bare H_2_Pc^39^ (First Column) and as Proposed for a H_2_Pc-Helium Solvation Complex (Second Column) That Leads to the Rotational Band Spectrum Shown in Figure 7 ^a^

	gas phase^39^	He-droplet^47^	sf
A″	299.03	66	4.5
B″	297.58	66	4.5
C″	149.30	50.3	3.0
A′	298.05	63	4.7
B′	297.83	63	4.7
C′	149.11	51.8	2.9

a Scaling factors (sf) for the moments of inertia calculated from the ratio of helium droplet data to gas phase data are listed in column 3.

Two observations need to be explained to further justify the two-component analysis shown in Figure 5, first the missing signature of free rotation within the sharp Gaussian peak and second the reversal of the solvent shift. As there is no evidence for the presence of a different doping species within the spectral range recorded in Figures 3, 4, and 5, the sharp Gaussian peak indicates an inhibition of the free rotation of the H_2_Pc-helium solvation complex. The reversal of the solvent shift must result from a reduction in the integrated dispersion interaction despite increasing amount of helium per droplet. All these features appear upon a significant change in the droplet source, namely, the change from gas phase expansion to liquid helium expansion.^36,43–45^ Under the given conditions, liquid helium expands from the normal fluid phase. Hydrodynamic effects such as capillary flow associated with uptake of angular momentum must be taken into account. According to the cylindrical symmetry of the hydrodynamic flux of liquid helium through the nozzle, the angular momentum preferably orients perpendicular to the axis of the helium flux, concomitant to the droplet jet axis, and thus be a cause of the observed optical anisotropy. Furthermore, angular momentum can change the droplet’s shape from spherical to ellipsoidal via centrifugal distortion as reported from X-ray and XUV-diffraction imaging experiments on large helium droplets.^28–31^ Moreover, a large enough angular momentum of a superfluid transforms into quantum vortices, which represent a strong local discontinuity in the helium density as visualized also by the above cited diffraction experiments.^48^

Any kind of aspherical deformation is synonymous with a redistribution of the helium to an on average more distant arrangement with respect to the center position. The same applies to an off-center position of the dopant, whether inside a spherical or an ellipsoidal droplet. Thus, an off-center position of the dopant, an aspherical droplet shape, or both in combination cause a reduction in solvent induced spectral shift as compared to the center position inside a spherical droplet. Implementing off center position and ellipticity to the line shape model of refs 25,26 these effects can be quantified. According to our model calculations an off center dopant’s position inside a spherical droplet (cf. Figure 8) or an ellipsoidal droplet shape with a centered dopant (cf. Figure 9) accounts for a reversal in the solvent shift for H_2_Pc even for increasing droplet size. For off-center dopant position in an ellipsoidal droplet the reversals shown separately in Figures 8 and 9 add up. The observed solvent shift reversal (cf. right ordinate in Figure 6) in the order of 10^–2^ cm^–1^ corresponds to realistic ellipsoidal droplet shapes and off-center dopant positions (cf. Figures 8 and 9).^28–31^

**Figure 8:**
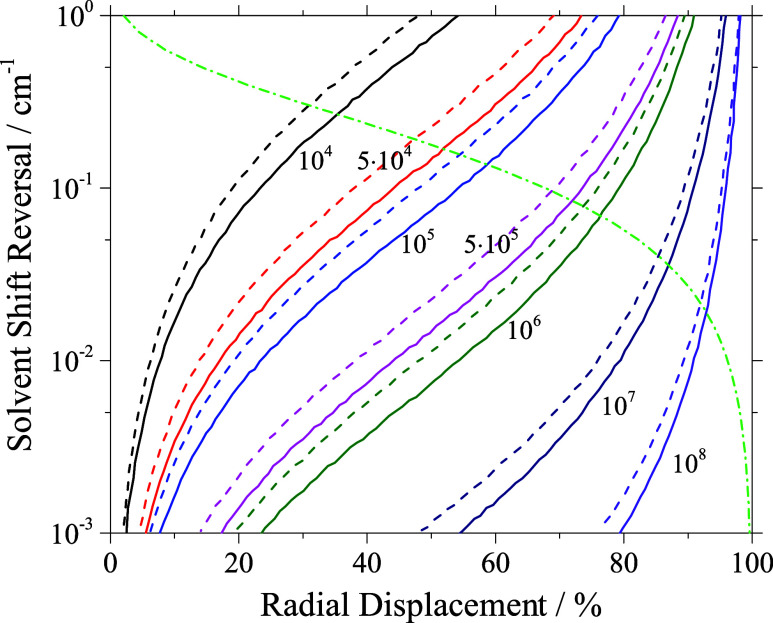
Solid lines: Solvent shift reversal calculated upon radial displacement of the H_2_Pc solvation complex for different droplet sizes as indicated by numbers of helium atoms. Dashed lines: Additional reversal induced by a gap in the helium density for dopant species trapped to a vortex (see text). Dash-dotted line: Boltzmann population distribution (abscissa) for solvent shift reversal as obtained for the droplet temperature of 0.37 K.

**Figure 9:**
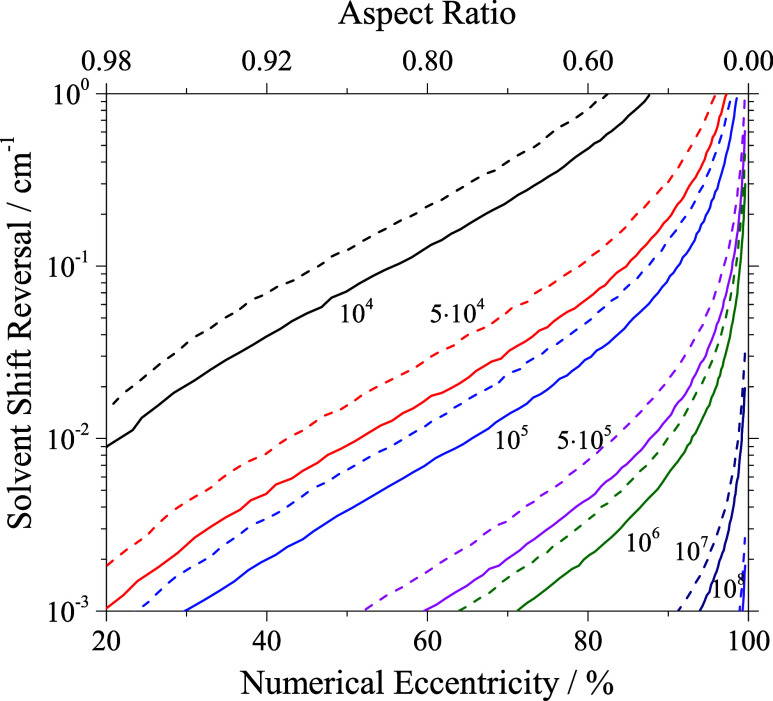
Solid lines: Solvent shift reversal calculated upon ellipsoidal deformation from spherically shaped droplets expressed by numerical eccentricity for droplet sizes as indicated by the number of helium atoms. In addition corresponding aspect ratio is added to the top axis. Dashed lines: Additional reversal induced by a gap in the helium density for dopant species simulating the trapping to a vortex (see text).

Note that the solvent shift reversal caused by off-center dopant position shown in Figure 8 exceeds that obtained for ellipticity of the droplet shape shown in Figure 9. However, the simulation for aspherical droplets depicted in Figure 9 consider perfectly elliptical droplet shapes whereas diffraction images reveal besides spherical and ellipsoidal in addition spheroidal, pill shaped, and dumbbell shaped droplets^49^ that cause larger displacement of the helium and, thus, larger solvent shift reversal. In Figure 9 the top axis shows the aspect ratio which is the ratio of the polar axis to the equatorial axis in the present case of the ellipse. The aspect ratio was also used in ref 49 to characterize the droplet shape. Since this parameter considers only two out of three inertial axes it can be calculated for any droplet, however, does not define a three-dimensional droplet shape.

In addition to the droplet size distribution provided by the helium droplet source and the fraction of singly doped droplets after passing through the pick-up oven, the droplet shape and dopant’s off-center shift add in so that at least four parameters instead of only two contribute to inhomogeneous line broadening at the electronic band origin. A fitting algorithm for line shape modeling considering all four parameters would not converge to a unique result. Nevertheless, the two additional parameters, namely droplets shape and dopant positioning account for the experimentally observed reversal in the solvent shift.

There is an additional conclusion that stems from the simulation of the influence of droplet shape and dopant position on the solvent shift. The dispersion model successfully applied for average droplet sizes below 10^5^ helium atoms considered only spherical droplets with the dopant in center position.^25,26^ Besides the difference in angular momenta gained for gas phase expansion, the gradients in the solvent shift reversal increases for decreasing droplet size (cf. Figures 8 and 9). Thus, smaller droplets are enforced to exhibit rather spherical shapes with the dopant in center position. A Boltzmann energy distribution calculated for the droplet temperature of 0.37 K added as dash-dotted green line to Figure 8 underlines the dominance of the strong gradient regime in the population distribution which justifies the restriction to spherical shapes and center position for droplets with less than 10^5^ helium atoms.

Finally, the sharp Gaussian peak needs to be evidenced by helium induced features that cause the sharp spectral width smaller than the expected rotational band contour of the solvation complex (cf. Figure 5 bottom row with the rotational band in Figure 7). Consequently, the rotation of the underlying H_2_Pc-solvation complex must differ from free rotation. The uptake of angular momentum by the droplets may generate vortices, a specific kind of angular momentum acquired by a superfluid.^33,48,50^ Trapped into or attached to a vortex^51–56^ the spatial isotropy for the dopant is broken and, thus, free rotation of the H_2_Pc-helium solvation complex is hindered. The hindered rotation may explain the sharp spectral width of the Gaussian signal contribution. Possibly, the sharp Gaussian peak position on the blue side of the doublet reflects the vortex induced reduction of helium density in the close vicinity of the trapped dopant. The dashed lines in Figures 8 and 9 have been calculated for a gap of roughly one helium layer between solvation complex and droplet. This is a simple and purely qualitative simulation of the effect of reduced helium density rather than a simulation that reflects that of a vortex truly. Nevertheless, capture by or attachment to a vortex is the most evident option for explaining the sharp Gaussian peak with its specific spectral features within the dispersion model. An alternative scenario, namely a location of the dopant almost at the surface of the droplet might be excluded for two reasons. First, the gradient of the solvent shift reversal depicted in Figure 8 drives the system toward the droplet volume as does the heliophilic character of H_2_Pc. Moreover, A positioning in the close vicinity of the surface can be expected to be accompanied by additional inhomogeneous line broadening. However, the two signal components do not show the corresponding increase of the line width.

## Conclusions

The line shape problem at the electronic band origin of H_2_Pc in helium droplets has found a convincing explanation, which represents an advance in the understanding of superfluid helium nanodroplets as a tool for the spectroscopic study of molecules, molecular aggregates and fundamental molecular dynamics at sub-Kelvin temperatures. New line shape studies, including the first investigation of optical anisotropy for H_2_Pc, reveal two signal components related to the solvation of the H_2_Pc molecule. One component is a sharp Gaussian peak that resides on the blue shoulder of a spectrally broader and asymmetric second component that may extend to the blue beyond the Gaussian peak. The helium-induced redshift of both signal components was reduced compared to the maximum measured for smaller droplet sizes. The interpretation of the two components could be made according to the shape and internal structure of helium droplets fully in line with results from X-ray and XUV diffraction experiments.^28–31^ Aspherical droplet shape, off center positioning of the dopant, and attachnment to votices^51–56^ are the features that serve for the experimental observations of a reversal in the solvent shift and the line splitting at the electronic band origin that is present at the electronic band origin of H_2_Pc doped into droplets generated from expansion of liquid helium. Even though on a first sight counterintuitive, all experimental observations for the line shape at the electronic band origin are fully consistent with London dispersion forces. The on a first sight surprising spectral shape is a fingerprint of the droplet’s shapes and of vortices. Both aspherical droplet shapes and vortices result from significant angular momentum taken up by droplets as to be expected from liquid helium expansion. Furthermore, the success of the dispersion model^25,26^ for line shapes of H_2_Pc in superfluid helium droplets generated from gas phase expansion can be taken as evidence for dominant presence of spherical droplets with the dopant in center position. A solid rebuttal of any ifs and buts requires an identification of similar spectral signatures for other dopant species which is subject of ongoing work in our laboratory.

## Supplementary Material



## Notes

Vacuum conditions under full operation of the droplet machine and on the interaction of the background gas with droplet size-dependent (PDF)
