# Supplementary material for: Fingerprint of Droplet Shape and Vortex in the Line Shape at the Electronic Band Origin of Phthalocyanine in Superfluid Helium Droplets
Source: ACS Phys Chem Au. 2025 Aug 7;5(5):467–77. doi: 10.1021/acsphyschemau.5c00018 (PMC12464779; doi:10.1021/acsphyschemau.5c00018)
Supplement: Supplementary file 1 [file pg5c00018_si_001.pdf]

Supporting Information for Publication Fingerprint of Droplet Shape and Vortex in the Line Shape at the Electronic Band Origin of Phthalocyanine in Superfluid Helium Droplets

Rupert P. M. Jagode, Alexander Scrimgeour, Florian Schlaghaufer, Johannes Fischer, and Alkwin Slenczka*

Institute for Physical and Theoretical Chemistry, University of Regensburg, 93053 Regensburg, Germany

E-mail: alkwin.slenczka@ur.de

*To whom correspondence should be addressed

Supplementary Material

Despite oil-free vacuum conditions, the background pressure under operating conditions must be evaluated for the effects on the passage of helium droplets of specific size. Background gas stems either from the helium droplet beam or from the hot pick-up oven.

Upon heating the pick-up oven to the maximum heating power of 10.5W applied in this study the pressure in the second vacuum chamber rose to $5 \cdot 10^{-8}$ mbar. Such a low level requires degasing of the H_2Pc sample for at least one day. The Poisson-statistical decrease of bare droplets by doping from the background gas along the beam axis is shown in Figure 1. The position of the laser beam with respect to the skimmer (at position 0) is marked by a vertical arrow. Note that these lines represent single droplet sizes out of a rather broad size distribution. The attenuation of droplet sizes close to or beyond 10^8 helium atoms is critical for the fluorescence detection system. At the considered level of background pressure caused by the hot pick-up unit limits the investigation to droplet sizes below 10^8 atoms. Note, that the assumed pressure results only from the maximum of heating power and is greatly reduced for the minimum heating power applied in the lower right section of the map shown in Figure 3 of the article.

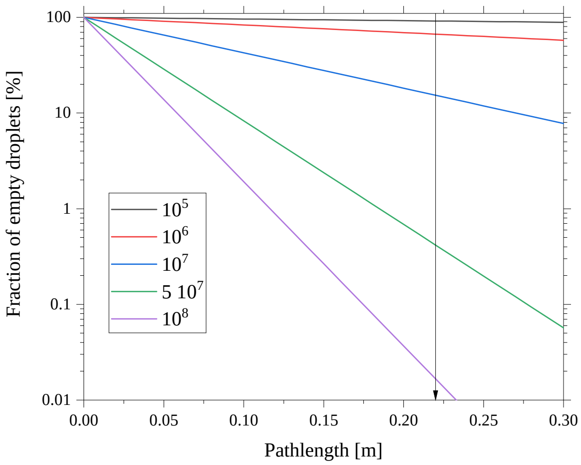

Figure 1: Fraction of droplets at different sizes (indicated by color) along the beam path from the skimmer (Pathlength = 0) to the laser beam (vertical arrow) that stay without doping from the background gas at a pressure of $5 \cdot 10^{-8}$ mbar.

Upon operation of the helium droplet source the background pressure rises to about $5 \cdot 10^{-7}$

mbar as peak value in the second vacuum chamber. This contribution to the background gas consists exclusively of atomic helium gas at an ambient temperature of about 300K. The main effect of this part of the background gas on the droplets is a sequence of pick-up collisions, the number of which can be calculated for certain droplet sizes (indicated by colors) according to Poisson statistics. Statistical distributions of collision numbers at the position of the skimmer (Pathlength about 0.11 m) are shown in Figure 2 for different droplet sizes as indicated. At the given loss of roughly 52 helium atoms per captured helium atom[1] the integral loss is safe below 5000 helium atoms that causes only a minor shift in the droplet size distribution.

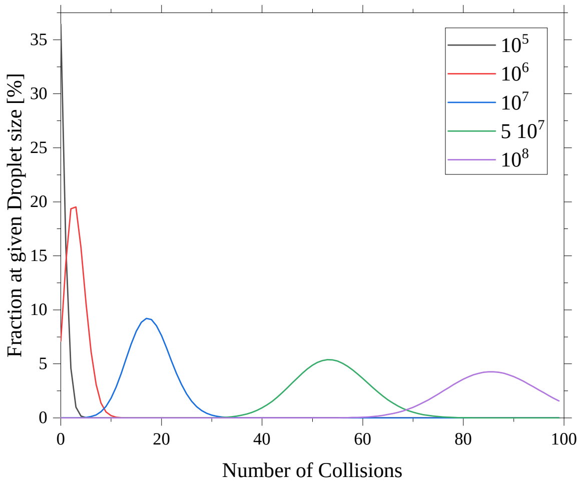

Figure 2: Distribution function of the number of collisions suffered by helium droplet at sizes indicated by color along a path of 0.22 m through helium gas at $5 \cdot 10^{-7}$ mbar.

Based on the dispersion model[2,3] the partial pressure of H_2Pc in the pick-up unit as function of the heating power could be deduced. The dispersion model applies to the spectra recorded for stagnation conditions of 20 bar and 11 K that are presented in the top row of Figure 3 in the article. The corresponding log-normal size distribution[4,5] has to be weighted by an appropriate Poisson distribution function for single particle pick-up within the given droplet size range. The particle density as parameter of the Poisson distribution function is the fitted parameter for each spectrum that can be transformed to the partial pressure. The resulting correlation of heating power to partial pressure is depicted in Figure 3.

As reported in Ref. 1 the average droplet sizes can be deduced from the intensity ratio of

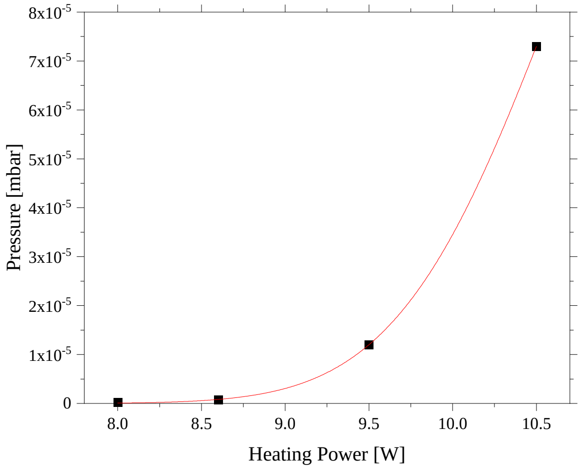

Figure 3: Heating power to partial pressure for H_2Pc in the pick-up oven deduced from spectra in the top row of Figure 3 in the article (cf. text). The red line is to guide the eye.

masses 8 amu, 12 amu, and 16 amu (cf. Fig. 8 in this Ref.). Corresponding figures given in Fig. 8 of this reference were confirmed in our experiment. Accordingly, the nozzle temperature can be translated to an average droplet sizes that spread from 10^4 atoms for 11 K to about $5 \cdot 10^7$ atoms for 6.5 K. In combination with the pick-up conditions, the optimum for single particle doping of H_2Pc is found for the spectra in Figure 3 of the article, which lie along the diagonal from top left to bottom right.

In summary the limiting factor for droplet sizes is the pick-up of foreign particles other than helium atoms or H_2Pc from the background gas. Under the given conditions the size range is limited to droplets of sizes below 10^8 helium atoms.

References

(1) Gomez, L. F.; Loginov, E.; Sliter, R.; Vilesov, A. F. Sizes of large He droplets. *The Journal of Chemical Physics* **2011**, *135*, 154201.

(2) Dick, B.; Slenczka, A. Inhomogeneous line shape theory of electronic transitions for molecules embedded in superfluid helium droplets. *The Journal of Chemical Physics* **2001**, *115*, 10206–10213.

(3) Slenczka, A.; Dick, B.; Hartmann, M.; Peter Toennies, J. Inhomogeneous broadening of the zero phonon line of phthalocyanine in superfluid helium droplets. *The Journal of Chemical Physics* **2001**, *115*, 10199–10205.

(4) Schilling, B. Molekularstrahlexperimente mit Helium-Clustern. Ph.D. thesis, Universität Göttingen, 1993; Max Planck Institut für Strömungsforschung, Report 14/1993.

(5) Lewerenz, M.; Schilling, B.; Toennies, J. A new scattering deflection method for determining and selecting the sizes of large liquid clusters of 4He. *Chemical Physics Letters* **1993**, *206*, 381–387.
